# Characterization of the complete chloroplast genome of *Euphorbia lathyris* (Euphorbiaceae), a traditional Chinese medicine

**DOI:** 10.1080/23802359.2020.1768169

**Published:** 2020-05-20

**Authors:** Xueyan Zhao, Qing Wang, Fangyuan Wang, Yan Li

**Affiliations:** Shaanxi Engineering Research Centre for Conservation and Utilization of Botanical Resources, Xi’an Botanical Garden of Shaanxi Province (Institute of Botany of Shaanxi Province), Xi’an, China

**Keywords:** *Euphorbia lathyris*, chloroplast genome, phylogenetic analysis

## Abstract

*Euphorbia lathyris* is a biennial herb plant in China. The seed is commonly used in well-known traditional Chinese medicine, and it has anti-tumor, *p*-glycoprotein and tyrosinase inhibitory activities. The length of circular chloroplast genome was 162,571 bp, containing a large single-copy region of 91,946 bp, a small single-copy region of 17,301 bp and two inverted repeat regions of 26,662 bp. The chloroplast genome contained 128 genes, including 85 protein-coding, 8 rRNA, and 35 tRNA genes. Phylogenetic tree analysis showed that *E. lathyris*, *E. kansui* and *E. esulaare* are closely related to each other.

*Euphorbia lathyris* is a biennial herb of *Euphorbia* (Euphorbiaceae). The dried seed of *E. lathyris* is used in traditional Chinese medicine and listed in the Chinese Pharmacopeia. It is widely applied to treat with edema (Chinese Pharmacopeia Committee 2010). Modern pharmacological experiments showed that *E. lathyris* had anti-tumor, *p*-glycoprotein and tyrosinase inhibitory activities (Appendino et al. 2001, 2003; Masamoto et al. 2003; Wang et al. 2011). In addition, *E. lathyris* seeds have a high oil content, and can be used for biodiesel production (Wang et al. 2011). Plant species are diverse and complex in Euphorbiaceae. In the present study, we characterize the complete chloroplast genome of *E. lathyris* and provide basic data for studying the phylogenetic relationships in Euphorbiaceae.

The fresh leaves of *E. lathyris* were sampled from Shaanxi Institute of International Trade and Commerce (34°29′N, 108°72′E; Shaanxi, China), and the voucher specimen (ZY190201) was deposited in the herbarium of the Xi’an Botanical Garden. Genomic DNA was extracted from the fresh leaves using the modified CTAB method (Doyle and Doyle 1987). Total DNA was used for the shotgun library construction and the subsequent high-throughput sequencing on the Illumina HiSeq 2500 Sequencing System.

After quality-trimmed and assembled using MITObim v1.8 (Hahn et al. 2013) with the reference sequence of *Euphorbia esula* (GenBank: NC_033910.1). The genome was annotated using software Geneious v9.0.2 (Biomatters Ltd., Auckland, New Zealand) by aligning with the reference chloroplast genome. The circular plastid genome map was completed using the online program OGDRAW (Lohse et al. 2013). The annotated chloroplast genome sequence has been deposited into the GenBank with the accession number MT241376.

The total plastome length of *E. lathyris* was 162,571 bp, with large single-copy (LSC, 91,946 bp), small single-copy (SSC,17,301 bp), and two inverted repeats (IRa and IRb; 26,662 bp each). The overall GC content was 35.7% (LSC: 32.8%; SSC: 30.5%; IRs: 42.4%) and the chloroplast genome contained 128 genes, including 85 protein-coding, 8 rRNA, and 35 tRNA genes.

In order to investigate the phylogenetic relationship of *E. lathyris*, the phylogenetic tree was constructed with MEG6 (Tamura et al. 2013) based on 12 complete chloroplast genome sequences of Euphorbiaceae and *Ilex cornuta* (Aquifoliaceae) (GenBank:NC_044416.1) as an outgroup (Figure 1). The results indicated that several plants of *Euphorbia* were placed as a branch, and *E. lathyris* has a close relationship with *E. kansui* and *E. esula*.

**Figure 1.**
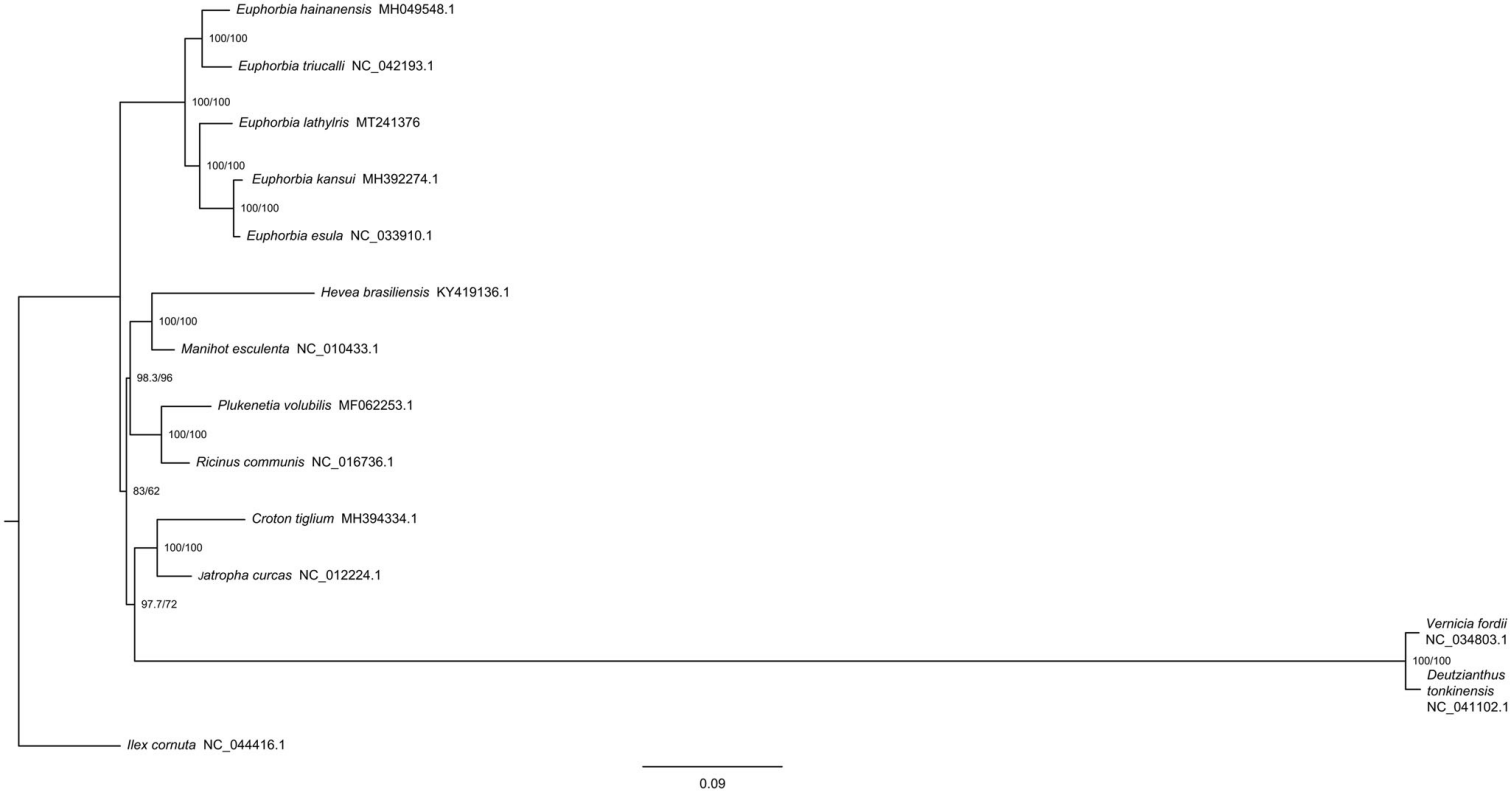
Phylogenetic tree based on 20 complete chloroplast genome sequences in Euphorbiaceae.

## Data Availability

The data that support the findings of this study are openly available in National Center for Biotechnology Information] at [https://www.ncbi.nlm.nih.gov/], reference number [MT241376].
